# Enhanced Detection of DNA Viruses in the Cerebrospinal Fluid of Encephalitis Patients Using Metagenomic Next-Generation Sequencing

**DOI:** 10.3389/fmicb.2020.01879

**Published:** 2020-08-12

**Authors:** Carmen F. Manso, David F. Bibby, Hodan Mohamed, David W. G. Brown, Mark Zuckerman, Jean L. Mbisa

**Affiliations:** ^1^Virus Reference Department, Public Health England, London, United Kingdom; ^2^Laboratorio de Virus Respiratorios e do Sarampo, Instituto Oswaldo Cruz/Fiocruz, Rio de Janeiro, Brazil; ^3^South London Specialist Virology Centre, King’s College Hospital NHS Foundation Trust, London, United Kingdom

**Keywords:** metagenomics, next-generation sequencing, encephalitis, DNA viruses, diagnostic techniques, bioinformatics

## Abstract

The long and expanding list of viral pathogens associated with causing encephalitis confounds current diagnostic procedures, and in up to 50% of cases, the etiology remains undetermined. Sequence-agnostic metagenomic next-generation sequencing (mNGS) obviates the need to specify targets in advance and thus has great potential in encephalitis diagnostics. However, the low relative abundance of viral nucleic acids in clinical specimens poses a significant challenge. Our protocol employs two novel techniques to selectively remove human material at two stages, significantly increasing the representation of viral material. Our bioinformatic workflow using open source protein- and nucleotide sequence-matching software balances sensitivity and specificity in diagnosing and characterizing any DNA viruses present. A panel of 12 cerebrospinal fluid (CSFs) from encephalitis cases was retrospectively interrogated by mNGS, with concordant results in seven of nine samples with a definitive DNA virus diagnosis, and a different herpesvirus was identified in the other two. In two samples with an inconclusive diagnosis, DNA viruses were detected and in a virus-negative sample, no viruses were detected. This assay has the potential to detect DNA virus infections in cases of encephalitis of unknown etiology and to improve the current screening tests by identifying new and emerging agents.

## Introduction

Encephalitis is a severe neurological syndrome defined by inflammation of the brain parenchyma in association with clinical evidence of neurological dysfunction (Tunkel et al., 2008). In Western countries, its annual incidence has been estimated to be 0.7–12.6 per 100,000 for adults and 10.5–13.8 per 100,000 for children (Jmor et al., 2008; Granerod et al., 2010c; Michael et al., 2010). Mortality rates range between 7 and 18%, and among the survivors, severe disability has been reported in up to 56% of the cases (Mailles and Stahl, 2009; Granerod et al., 2010a; Thakur et al., 2013). Encephalitis has multiple etiologies and pathogeneses. Viruses have been reported as the most common etiological agents, causing 20–50% of the encephalitis cases (Glaser et al., 2006; Granerod et al., 2010b; Ambrose et al., 2011). Immune-mediated etiology has been increasingly recognized as the second most common cause of the disease (Gable et al., 2009; Granerod et al., 2010a; Scheer and John, 2016). Strikingly, in more than 50% of cases, the etiology remains undetermined (Glaser et al., 2006; Florance et al., 2009; Gable et al., 2009; Venkatesan et al., 2013).

The “gold standard” diagnostic test is the pathologic examination and testing of brain tissue, however, this is rarely done ante-mortem due to potential morbidity associated with an invasive neurosurgical procedure. The most frequently used diagnostic procedures include PCR detection of causative pathogens in cerebrospinal fluid (CSF) and blood, serological testing for specific antibodies in blood and CSF, and occasionally pathogen culture (Solomon et al., 2007). Herpes simplex virus type 1 (HSV-1), varicella-zoster virus (VZV), and any of a number of *Enterovirus* species are identified by CSF PCR in 90% of the cases where a viral pathogen is identified (Solomon et al., 2012). Other members of the *Herpesviridae* are commonly detected in encephalitis cases – HSV-2, Epstein-Barr virus (EBV), cytomegalovirus (CMV), and human herpesvirus types 6 and 7 (HHV-6 and -7) – in addition to viruses from diverse families including *Adenoviridae*, *Paramyxoviridae*, *Orthomyxoviridae*, *Polyomaviridae*, *Rhabdoviridae*, *Parvoviridae*, *Astroviridae*, *Pneumoviridae*, *Retroviridae*, several arboviruses from the *Flaviviridae*, *Bunyaviridae*, and *Reoviridae*, and both zoonotic and non-zoonotic members of the *Togaviridae*, and *Arenaviridae* (Palacios et al., 2008; Mailles and Stahl, 2009; Quan et al., 2010; Chan et al., 2014; Fok et al., 2015; Naccache et al., 2015; Haley and Atwood, 2017; Crawshaw et al., 2018; Mehta et al., 2018; Vidal et al., 2019). This list is not exhaustive.

Existing diagnostic methods, although somewhat successful for known viruses, are limited by their high specificity when employed to detect genetically divergent, unknown, or unexpected viruses that might be present in the sample. Together with the large and expanding number of pathogens reported to be capable of causing encephalitis (Granerod et al., 2010c; Gurav et al., 2010; Benjamin et al., 2011; Solomon et al., 2012; Woolhouse et al., 2012; Woolhouse and Adair, 2013; Fok et al., 2015; Hoffmann et al., 2015; Kennedy et al., 2017), it is perhaps unsurprising that so many cases have inconclusive etiology.

Metagenomics, the direct and sequence-agnostic analysis of all genetic material within a sample, coupled with the massively parallel sequencing capabilities of metagenomic next-generation sequencing (mNGS) represents a potential breakthrough in the diagnosis of encephalitis and has led to the discovery of a large number of novel and/or unexpected viral agents of disease (Tan et al., 2013; Phan et al., 2015; Kawada et al., 2016; Kang et al., 2017; Morfopoulou et al., 2017; Bukowska-Ośko et al., 2018; Oechslin et al., 2018; Piantadosi et al., 2018; Eibach et al., 2019; Wilson et al., 2019).

Nonetheless, viral mNGS is a challenging approach due the low relative abundance of virus-derived material in clinical specimens compared to host-derived material. Improving this ratio is key to achieving a sufficient amount of viral reads to allow reliable detection and accurate identification of viruses in a sample (Chan et al., 2014; Hall et al., 2014; Kohl et al., 2015; Lewandowska et al., 2015; Bukowska-Ośko et al., 2017). Selective depletion of the ribosomal RNA (rRNA) fraction followed by DNAse digestion resulted in a significant methodological improvement in the mNGS protocol for RNA viruses previously developed in our laboratory (Manso et al., 2017). However, effective enrichment of viral DNA has proven to be more challenging due to the lack of differential motifs between human and viral DNA that allow depleting the former without affecting the number of copies of the latter in the sample.

Here, we describe a DNA mNGS protocol focused on increasing the relative abundance of viral DNA at two stages: (i) before extraction, by performing selective lysis of mammalian cells with digitonin, a specific steroidal saponin used by researchers to manipulate cell membranes (Hannah et al., 1998; Jamur and Oliver, 2010), followed by DNAse digestion of host genomic DNA, and (ii) after generating metagenomic libraries, by size selection of library fragments. These two approaches notably improve the detection and characterization of DNA viruses in the Clinical Virology Multiplex I panel (CVM panel) and clinical CSF samples.

## Materials and Methods

### Ethics Statement

All experiments were performed in accordance with the “Guidance on Conducting Research in Public Health England” (Version 3, October 2015; Document code RD001A). This study involved the use of archived, residual samples that were collected as part of a prospective etiological study on encephalitis in the UK with approval from the North and East Devon Multicenter Research Ethics Committee (05/Q2102/22). The samples were anonymized by removal of any patient identifiable information and assignment of a non-specific project number prior to genetic characterization.

### Clinical Virology Multiplex I Panel (CVM Panel)

A lyophilized reagent comprising 11 DNA viruses known to cause encephalitis was obtained from the National Institute of Biological Standards and Controls (Potters Bar, UK, catalog number 15/130-xxx). Prior to extraction, the reagent was resuspended in 1 ml of negative CSF sample. The following viruses were included in the panel: adenovirus serotype 2 (AdV-2), BK and JC polyomaviruses (BKPyV and JCPyV, respectively), HSV-1, HSV-2, CMV, EBV, VZV, HHV-6a and b, and Parvovirus B19 (PV B19). Further information as to their characteristics can be found on the NIBSC website^1^ and in studies by Doris et al. (2015).

### Clinical CSF Samples

A total of 12 CSF samples from patients suffering from acute viral encephalitis, previously characterized by routine diagnostic testing.

### Digitonin-DNAse Treatment of CSF Samples

Plasma membranes of cells present in 200 μl CSF were permeabilized by adding digitonin (Sigma Aldrich, Poole, UK) to a final concentration of 25–100 μg/ml and incubating at 37°C for 5 min, followed by the addition of 2 U of Turbo DNAse enzyme and Turbo DNAse buffer (both ThermoFisher, Dartford, UK) to a final concentration of 1X. Digests were incubated at 37°C for 10 min, followed by immediate extraction.

### Nucleic Acid Extraction and Library Preparation

A total of 200 μl of either untreated or digitonin-DNAse-treated CSF was extracted using PureLink Viral RNA/DNA Mini Kit (Invitrogen, Renfrewshire, UK) following the manufacturer’s specification but omitting carrier RNA. Concentrations of dsDNA in extracts were determined using the Quant-it dsDNA HS Assay Kit on a Qubit 3.0 fluorometer (both Invitrogen).

Sample extracts were diluted to 0.2 ng/μl where possible; extracts with lower DNA concentrations were used without dilution. DNA libraries were prepared from 5 μl DNA using the Nextera XT DNA library prep kit (Illumina, Cambridge, UK) according to the manufacturer’s instructions.

The standard protocol for the clean-up of libraries used AMPure XP beads (Beckman Coulter, High Wycombe, UK) at the recommended 1.8X bead ratio. The effects of single and double clean-up steps and the use of 0.85X bead concentration were investigated. Following clean-up, libraries were analyzed for size distribution using the High Sensitivity DNA Kit on a 2100 Bioanalyzer Instrument (both Agilent, Stockport, UK) and were quantified using Qubit, as described above.

Batches of four libraries labeled with different indexes were pooled; within each pool, each component library contributed the same total mass. Pools were further quantified by Qubit, as described above and diluted to a final concentration of 2 nM before being denatured with 0.2 N sodium hydroxide for 2 min, diluted in kit reagent HT1 to produce a 20 pM solution and then further diluted to 7.9 pM. Of this library pool dilution, 600 μl was loaded onto a MiSeq cartridge. Sequencing was performed on an MiSeq instrument using the MiSeq Reagent Kit V2 (300 cycles; both Illumina) according to the manufacturer’s guidelines.

### Real-Time PCR

The relative abundance of human material in sample extracts was evaluated by real-time PCR using primer and probe sets targeting *c-myc* (Schroeder and Nitsche, 2010) and β-globin (Lo et al., 1998). Reactions were performed using the KAPA Probe Fast Universal Kit (Roche, Burgess Hill, UK) according to the manufacturer’s instructions.

### Data Analysis

Adapters and poor-quality terminal bases were removed from paired-end FASTQ files with Trimmomatic v0.39 (Bolger et al., 2014; RRID:SCR_011848); followed by removal of duplicates and low-complexity reads using PRINSEQ (Schmieder and Edwards, 2011; RRID: SCR_005454) with an entropy cut-off of 70 and all de-duplication options selected. Cleaned FASTQs were mapped with PALADIN (Westbrook et al., 2017) to a database comprising the RefSeq viral protein sequences downloaded from the /refseq/release/viral directory within the NCBI ftp repository (located at ftp://ftp/ncbi.nlm.nih.gov) supplemented with the NCBI RefSeq human protein sequences (/refseq/H_sapiens/mRNA_Prot). NCBI taxonomy files (/pub/taxonomy/taxdmp.zip) were used to map taxon IDs to each reference (Brister et al., 2015; RRID:SCR_003496). Hits were considered only for those mapping results having an e-score below 10^−10^. For each hit, taxon IDs for the mapped target itself and all of its parental taxonomic divisions were obtained by iteratively querying the nodes.dmp file of taxdmp.zip; counters were incremented for all taxon IDs common to both ends of paired-end reads. Outputs were limited to viruses within taxonomic divisions known to infect humans (Woolhouse et al., 2012; Woolhouse and Adair, 2013).

In the second analysis stage, cleaned FASTQs were mapped with BWA MEM (Li, 2013; RRID:SCR_010910) to RefSeq genomes of those viruses having over 1.5 reads per million assigned by PALADIN. To visualize detection of diverse genome fragments within a target virus, mapped reads were binned by the percentile within the genome length of their mapped starting position (the POS field in the SAM files).

## Results

### Digitonin-DNAse Treatment Depletes Human DNA From CSF Extracts

The effect of digitonin-DNAse treatment on human DNA concentrations in nucleic acid extracts was investigated by treating a virus-negative CSF sample with 25, 50, 75, or 100 μg/ml digitonin followed by DNAse digestion. Real-time PCR against c-myc and β-globin showed greater than 99% reduction in human material in both cases, with Qubit spectrophotometry showing an approximate 90% reduction (Table 1) at a concentration greater than 50 μg/ml. Similar analysis of the CVM panel resuspended in a virus-negative CSF sample treated with 50 μg/wml digitonin and DNAse showed a reduction of 95–99% human material compared to controls. This protocol enhancement was applied to four CSF samples from patients diagnosed with viral encephalitis. In three of the four, a reduction in human material of up to 98% was obtained. In the fourth sample, no reduction was observed, although the initial concentration was very low.

**Table 1:** Reduction of human DNA in cerebrospinal fluid (CSF) extracts following digitonin-DNAse treatment of three sample sets.

	Digitonin conc. (μg/ml)	Threshold cycle (C_t_)		Total DNA conc. (ng/μl)
		c-myc	β-globin	
Control CSF	0	31.5	32.2	1.23
	25	35.5	36.4	0.15
	50	>40	>40	<0.10
	75	36.4	>40	0.13
	100	>40	>40	0.17
Control CSF spiked with CVM I Panel	0	30.2	29.4	1.95
		30.3	29.4	2.12
	50	35.9	35.9	<0.10
		35.7	35.8	0.10
Patient CSF samples	0	29.0	28.6	1.6
		28.4	28.2	1.8
		27.3	27.5	3.45
		33.6	34.2	<0.10
	50	32.1	32.2	0.22
		33.9	34.0	<0.10
		30.3	30.0	0.56
		32.5	33.5	<0.10

DNA concentrations were determined by Qubit analysis.

### Digitonin-DNAse in Combination With AMPure Size Selection Enhances Virus Detectability in CSF Extracts

Libraries prepared from duplicate extracts of both control and digitonin-DNAse treated CVM panel aliquots were sequenced on a single MiSeq run, yielding an average of 2.6 million paired-end reads per sample. With the exception of HSV-2, which showed a slight reduction in relative read count compared to the control, all viruses showed an increase (Table 2). BKPyV was exceptional in showing an average 60x increase. VZV, JCPyV, and adenovirus (AdV) showed increases of 30x, 28x, and 15x, respectively. The remaining panel viruses showed 1.6–2.6x increases. Strikingly, PV B19 was undetectable in the control libraries, but three reads per million (*rpm*) were detected in the two libraries following digitonin-DNAse treatment.

**Table 2:** Summary of total number of reads and reads matching viral targets expressed per 1 million QC-filtered reads in the Clinical Virology Multiplex I panel, showing the effects of duplicate digitonin-DNAse and size-selection treatments.

Virus	No treatment	Digitonin	Size-selection	Digitonin and size-selection
AdV	41 29	458 476	33 31	925 1,003
PV B19	-	3 3	-	5 8
JCPyV	28 30	751 778	32 25	1,434 1,552
BKPyV	24 55	1,948 2,083	40 54	4,067 4,146
HSV-1	8 12	21 29	12 6	27 56
HSV-2	57 95	74 61	69 62	89 88
VZV	11 20	432 386	14 14	864 808
HHV-6a	63 46	84 104	56 60	113 113
HHV-6b	54 51	98 134	51 52	123 203
CMV	59 51	81 87	55 63	161 192
EBV	39 44	84 113	47 36	119 150
Total reads (×10^6^)	1.27 1.21	2.39 2.85	2.63 2.45	2.58 2.68

**Figure 1:**
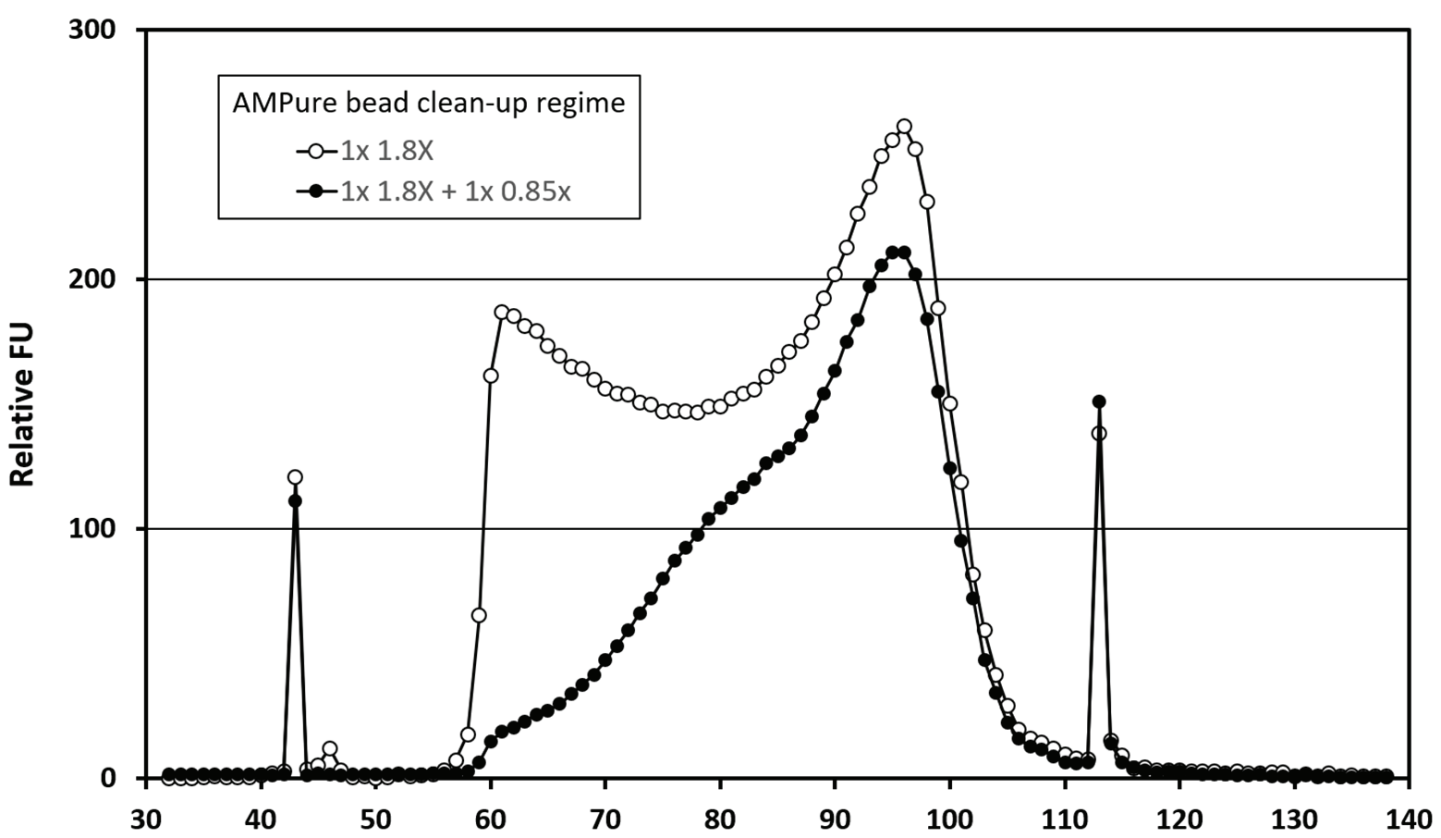
Bioanalyzer traces showing the effect of AMPure bead ratio on library fragment size distribution. Traces represent analyses of a pool of four Clinical Virology Multiplex I panel (CVM panel) libraries, two of which were digitonin-DNAse treated and two untreated. The pool was initially cleaned using a 1.8X AMPure bead ratio (open circles), before being cleaned a second time using a 0.85X AMPure bead ratio (closed circles). The *x*-axis time variable correlates with library fragment size – earlier fragments are shorter than later ones. The peaks at 43 and 113 s are internal control fragments of known molecular weight.

The four libraries from the previous analysis were subjected to a second clean-up step at an AMPure bead ratio of 0.85X. This step selectively removes shorter fragments from the libraries (Figure 1). The rightmost columns of Table 2 show a modest effect of up to 1.3x on the detection of viral reads in the control samples and a 1.3–2.5x effect upon the digitonin-DNAse treatment libraries. With both enhancements combined, BKPyV showed the highest increase in read frequency (131x). VZV, JCPyV, and AdV showed 61x, 59x, and 32x increases, respectively, with the remaining herpesviruses showing a combined increase of 1.3–6.2x.

### Application of the Enhanced Protocol to CSF Samples From Encephalitides

A series of 12 CSF samples from acute encephalitis patients was tested together with negative human plasma (NHP) and water controls, using the enhanced protocol incorporating the digitonin-DNAse and size selection modifications. With conventional diagnostics, a DNA virus etiology was established in nine of the patients and excluded in one (Table 3). In samples from the remaining two patients (nos. 1 and 11), the presence of AdV and BKPyV (respectively) was provisional; the real-time PCR curves emerged at a cycle beyond the established limit of detection of the assay. Four samples were multiplexed on each MiSeq sequencing run, giving approximately 3–5 million reads per sample.

**Table 3:** Metagenomic Next-Generation Sequencing (NGS) results for 12 CSF samples derived from patients with encephalitis, together with two control samples.

Sample	Routine diagnosis	Total reads (×10^6^)	Virus detected	Reads per million	Mapped reads	Genome percentiles
1^†^	AdV	4.73	AdV	0	0	0
			HPV10	229	2,222	100
2	VZV	7.12	VZV	21	616	87
3	VZV	3.66	VZV	56	463	67
4	VZV	4.94	VZV	0	214	3
			EBV	16	103	67
5	HHV-6	4.44	HHV-6	0	11	9
			EBV	8.6	59	33
6	VZV	3.52	VZV	98	595	87
7	JCPyV	2.98	AdV	3.0	0	0
			JCPyV	16	71	34
			EBV	2.7	8	5
8	JCPyV	3.32	JCPyV	21	314	56
			AdV	2.7	0	0
9	VZV	3.23	VZV	50	424	79
10^‡^	β-glucan^+^	3.43	None			
11^†^	BKPyV	3.33	BKPyV	0	0	0
			EBV	8.7	47	32
			TTV	29	327	47
12	VZV	4.94	VZV	161	1,714	100
NHP	-	1.51	HHV-6	3.3	14	8
H_2_O	-	0.20	None			

For each virus detected by the PALADIN component of the pipeline, the number of reads per million total reads is given, alongside the outputs of the downstream BWA mapping process.

† Routine diagnostic qPCR results for samples 1 and 11 were beyond their limits of detection and are possible artifacts.

‡ This result is indicative of the presence of a fungal pathogen.

**Figure 2:**
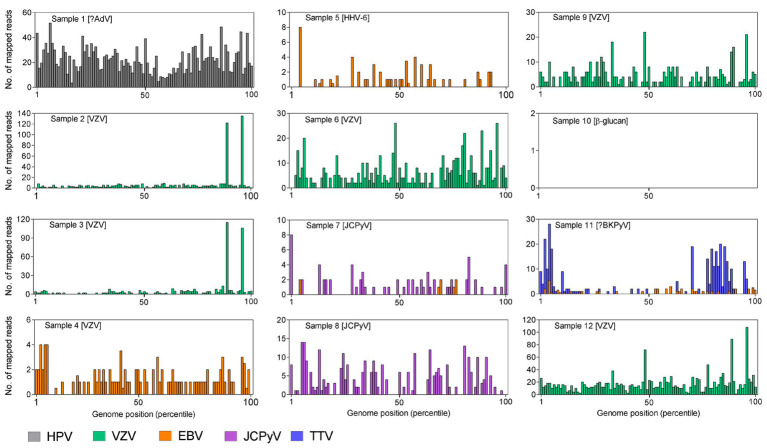
BWA mapping against the reference sequence of viruses detected by PALADIN in the 12 datasets from clinical cerebrospinal fluid (CSF) samples. Results are expressed as reads per genome percentile. No DNA viruses were detected in sample 10. In brackets are the diagnoses made by the originating laboratory using routine testing.

In seven of the diagnoses (five of VZV and two of JCPyV), the metagenomic analysis gave a strong corroborating signal with 16–161 *rpm* (Table 3, nos. 2, 3, 6–9, and 12). A sixth VZV diagnosis (no. 4) gave only EBV hits by PALADIN at 16 *rpm*, mapping to a broad range of genomic regions, suggesting possible initial mis-diagnosis (Figure 2). Mapping this sample’s FASTQ files to the VZV reference genome gave over 200 hits, but these were almost entirely targeting two short regions of VZV; only three regions in total had any mapping at all.

Similarly, EBV was the only virus detected in a sample initially diagnosed with HHV-6 infection (no. 5 in Table 3), with 8.6 *rpm* detected by PALADIN. Sample no. 11 (one of the two samples with a late diagnostic PCR – BKPyV in this instance), gave 8.7 *rpm* for EBV. Both samples’ read sets again mapped to diverse regions of the EBV genome, whereas in the former, only 11 reads mapped to the HHV-6b genome across nine percentiles. The latter sample also had a relatively high number (22 *rpm*) of torque teno virus (TTV) reads detected, also mapping across genome percentiles, although no higher resolution identification than the genus alphatorquevirus was possible. The second late-cycle diagnosis, for AdV (sample no. 1), gave a very high number of reads for human papillomavirus type 10 (HPV10), sufficient to assemble an entire genome (data not shown).

The final sample (no. 10) was positive for the β-glucan biomarker, suggesting the presence of a fungal pathogen (Lyons et al., 2015), and no viral targets were detected in this sample. Secondary AdV detections were made by PALADIN analysis in the two JCPyV-positive samples (nos. 7 and 8). However, reference mapping of these targets indicated that these were false positives, with zero AdV-mapping reads in either sample. An additional PALADIN detection of EBV in sample 7 corresponded to a total of just eight EBV-mapping reads.

In the two controls, the water control gave few reads, of which none derived from a DNA virus, whereas HHV-6b was detected in the NHP control at a rate of 3.3 *rpm*, with 14 mapped reads across eight genome percentiles.

## Discussion

Effective treatment of many forms of encephalitis relies upon a prompt response, with delays often leading to devastating consequences. The high number of cases in which the etiological agent remain undiagnosed highlights the need for improved diagnostic methods. The use of unbiased, sensitive and cost-effective metagenomic NGS assays to sequencing the total RNA and DNA in a sample represents a potential breakthrough in the diagnosis of infectious encephalitis.

In this study, we present an mNGS protocol that allows enhanced detection and characterization of DNA viruses in CSF samples, overcoming the challenges of low target abundance through the use of digitonin-DNAse treatment and AMPure bead-based size-selection of library fragments. Up to 99% of the human DNA was removed by this method – more than methods exploiting differential methylation between host and viral genomic material (Feehery et al., 2013; Oyola et al., 2013; Thoendel et al., 2016). Concomitantly, virus read frequencies were enhanced by up to nearly 60-fold. These values compare favorably with conventional methods of viral enrichment, where the depletion of human material either led to only modest increases in viral read frequency (Hall et al., 2014), or improvements dependent upon either the virus and techniques used (Kohl et al., 2015), or high nucleic acid input quantities (Conceição-Neto et al., 2015; Parras-Moltó et al., 2018). This mirrors our experience with these physicochemical methods in that the recovery of some viruses can be enhanced, but this is invariably at the expense of others. Although other saponins have been successfully applied to pathogen metagenomics (Hasan et al., 2016), the variability of commercial saponin products reduces its potential for use in clinical applications.

A second enhancement followed the observation that in DNA libraries prepared from digitonin-DNAse treated samples, fragments of human origin had a size distribution considerably shorter than viral fragments (data not shown). We hypothesize that much of this material represents DNAse-hypersensitive mononuclosomes (Schwartz et al., 2019). Size-selection of libraries derived from both virus panels and clinical samples with a low ratio of AMPure beads resulted in a further increase in the frequencies of viral reads, as well as a greater representation of reads from diverse genomic regions of the detected viruses. The PV B19 genomes in the CVM panel gave low *rpm* values, and then only after digitonin treatment, perhaps reflecting a low copy number in the CVM panel and/or the single-stranded nature of its genome. AMPure bead-based size selection is routinely used to selectively remove adapter dimers from library preps, but other than a recent VIDISCA paper (Edridge et al., 2019), this is the first time it has been used to perform the enrichment of viral DNA fragments on library preps from CSF samples.

The enhanced mNGS workflow was challenged using a panel of CSF samples from patients suffering from encephalitis, previously diagnosed by routine diagnostic tests. Sequencing data were blindly analyzed, and achieved concordant results in seven of the nine samples with a definitive diagnosis. In the two discordant samples, mNGS clearly detected a different viral species within the *Herpesviridae* family from that originally diagnosed, both through PALADIN and through reference mapping. Unfortunately, retrospective confirmatory laboratory tests could not be performed owing to a lack of remaining sample, and the cause of the discrepancies remains unclear. Reads from both samples mapped to their originally diagnosed viruses (HHV-6 and VZV), but in both cases, the number and distribution of hits were both much lower than for those detected by PALADIN.

In one of two CSF samples with an unconfirmed diagnosis by routine testing, the presence of EBV and TTV was identified by mNGS; TTV has been recently detected in CSF samples from encephalitis patients, and EBV is a well-established cause (Kang et al., 2017; Eibach et al., 2019). In the second, mNGS was able to assemble a complete genome of HPV10, an alphapapillomavirus exclusively associated with cutaneous lesions (Cubie, 2013), and hence most likely to represent a skin flora contaminant arising from lumbar puncture. A final sample had a putative diagnosis of a fungal agent, and our mNGS assay detected no viral reads.

The impact of hits caused by read mis-assignment or reads from reagents and environmental contaminants was initially minimized by filtering the mapping results both by e-score and by limiting outputs to viruses within taxonomic divisions known to infect humans (Woolhouse et al., 2012; Woolhouse and Adair, 2013). Notwithstanding these filters, in two JCPyV-positive samples, a low number of AdV reads were detected. In one of the two, EBV was also detected. Secondary reference mapping analysis revealed the AdV detections to be false positives and the EBV attribution to be doubtful, owing to the low number of both hits and mapped reads, in contrast with the high values from the true positive JCPyV outputs. These data support the use of multiple bioinformatic tools with diverse algorithmic natures, a principle that has been repeatedly shown to improve the accuracy of metagenomic analyses (Lin and Liao, 2017; McIntyre et al., 2017). In a recent paper (Miller et al., 2019), a group from San Francisco proposed having at least three viral reads spanning at least three non-overlapping regions of the most closely matched reference sequence as a requirement to report a pathogen detected by metagenomics. While in the light of VZV data from sample 4 and the hits at up to 5 *rpm* in both samples and the NHP control we would advocate more stringent corroborating metrics within our metagenomic assays, the application of thresholds at a level enabling discrimination between true and false positive detections, while retaining a useful sensitivity remains largely empirical. Hence, in all cases, formal diagnosis necessitates confirmation with pathogen-specific assays (Granerod et al., 2010b; Brown et al., 2018), although the diagnosis of HHV-6 in sample 5 could be dependent upon the testing algorithm. It should be noted, however, that demonstrating that a detected agent is causative can be problematic, particularly in cases where a novel agent is discovered (Tan et al., 2013; Phan et al., 2015, 2016; Bukowska-Ośko et al., 2018; Eibach et al., 2019).

The level of agreement between our mNGS results and routine diagnostics compares favorably to those of other authors. For example, a similar Swiss study recently reported metagenomic analyses of six CSF samples with a DNA virus diagnosis, of which only one was reliably concordant with prior diagnostics. In the remainder, the signal-to-noise ratios were insufficient to consider the metagenomic information valid (Oechslin et al., 2018). In another recent study using the VIDISCA-NGS technique, CSF samples were tested in which the presence of herpesvirus had been previously diagnosed by routine qPCR test. Digestion of target material during DNase treatment presented a problem and as a result, virus was detected in just one of the DNAse treated samples. Less than 30% of the non-DNAse treated samples gave a signal, and only then in high viral load samples (Edridge et al., 2019).

In contrast, the San Francisco group reported a strong concordance between routine and mNGS results. The study evaluated the accuracy of a mNGS assay for detection of pathogens causing encephalitis, including 26 DNA virus positive and 19 DNA virus negative samples previously tested by qPCR assay, observing a 89.8% accuracy. This value increased to 92.4% when repeat testing of discrepant samples was performed (Miller et al., 2019).

To conclude, digitonin-DNAse treatment can effectively improve the ratio of viral to host DNA in CSF samples. The proportion of viral reads can be further improved by size-selecting libraries prepared from digitonin-DNAse treated samples. The use of effective enrichment methods allows more samples to be multiplexed per sequencing run, thus reducing costs and making the mNGS approach more economical in the clinical setting. By applying only moderately advanced bioinformatic tools, the presence of DNA viruses can be successfully identified in the resulting mNGS datasets. Thus, in conjunction with a parallel RNA virus method (e.g., adapted from Manso et al., 2017), this proposed mNGS assay has the potential to help detect viral causative agents from the high number of encephalitis cases with unknown etiology and to be used as a second-line test to current target-specific assays. The increased accessibility of NGS technologies in clinical microbiology laboratories and the ever-decreasing costs of running these tests should make this a reality. Furthermore, mNGS will spur improvements in the current screening tests by identifying new and emerging etiological agents which could be later incorporated into the target-specific first-line tests.

## Data Availability Statement

The raw data supporting the conclusions of this article will be made available by the authors, without undue reservation.

## Ethics Statement

All experiments were performed in accordance with the “Guidance on Conducting Research in Public Health England” (Version 3, October 2015; Document code RD001A). This study involved the use of archived, residual samples that were collected as part of a prospective etiological study on encephalitis in the UK with approval from the North and East Devon Multicenter Research Ethics Committee (05/Q2102/22). The samples were anonymized by removal of any patient identifiable information and assignment of a non-specific project number prior to genetic characterization.

## Author Contributions

CM carried out research in the lab. DFB carried out the bioinformatic data analysis. HM contributed to lab research. MZ performed sample primary diagnostic and assembled the CSF clinical sample set. CM, JM, and DWGB conceived of the project. CM, DFB, and JM conceived of lab methods. JM supervised the project. DFB and CM wrote the manuscript. All authors contributed to the article and approved the submitted version.

### Conflict of Interest

The authors declare that the research was conducted in the absence of any commercial or financial relationships that could be construed as a potential conflict of interest.

## Footnotes

^1^www.nibsc.org/documents/ifu/15-130-xxx.pdf

## References

[ref1] Ambrose H. E. Granerod J. Clewley J. P. Davies N. W. S. Keir G. Cunningham R.. (2011). Diagnostic strategy used to establish etiologies of encephalitis in a prospective cohort of patients in England. J. Clin. Microbiol. 49, 3576–3583. 10.1128/JCM.00862-11, PMID: 21865429 PMC3187347

[ref2] Benjamin L. A. Lewthwaite P. Vasanthapuram R. Zhao G. Sharp C. Simmonds P.. (2011). Human parvovirus 4 as potential cause of encephalitis in children, India. Emerg. Infect. Dis. 17, 1484–1487. 10.3201/eid1708.110165, PMID: 21801629 PMC3381555

[ref3] Bolger A. M. Lohse M. Usadel B. (2014). Trimmomatic: a flexible trimmer for Illumina sequence data. Bioinformatics 30, 2114–2120. 10.1093/bioinformatics/btu170, PMID: 24695404 PMC4103590

[ref4] Brister J. R. Ako-Adjei D. Bao Y. Blinkova O. (2015). NCBI viral genomes resource. Nucleic Acids Res. 43, D571–D577. 10.1093/nar/gku1207, PMID: 25428358 PMC4383986

[ref5] Brown J. R. Bharucha T. Breuer J. (2018). Encephalitis diagnosis using metagenomics: application of next generation sequencing for undiagnosed cases. J. Inf. Secur. 76, 225–240. 10.1016/j.jinf.2017.12.014, PMID: 29305150 PMC7112567

[ref6] Bukowska-Ośko I. Perlejewski K. Nakamura S. Motooka D. Stokowy T. Kosińska J.. (2017). Sensitivity of Next-Generation Sequencing metagenomic analysis for detection of RNA and DNA viruses in cerebrospinal fluid: the confounding effect of background contamination. Adv. Exp. Med. Biol. 944. 10.1007/5584_2016_42, PMID: [Epub ahead of print] 27405447

[ref7] Bukowska-Ośko I. Perlejewski K. Pawełczyk A. Rydzanicz M. Pollak A. Popiel M.. (2018). Human pegivirus in patients with encephalitis of unclear etiology, Poland. Emerg. Infect. Dis. 24, 1785–1794. 10.3201/eid2410.180161, PMID: 30226156 PMC6154136

[ref8] Chan B. K. Wilson T. Fischer K. F. Kriesel J. D. (2014). Deep sequencing to identify the causes of viral encephalitis. PLoS One 9:e93993. 10.1371/journal.pone.0093993, PMID: 24699691 PMC3974838

[ref9] Conceição-Neto N. Zeller M. Lefrère H. De Bruyn P. Beller L. Deboutte W.. (2015). Modular approach to customise sample preparation procedures for viral metagenomics: a reproducible protocol for virome analysis. Sci. Rep. 5:16532. 10.1038/srep16532, PMID: 26559140 PMC4642273

[ref10] Crawshaw A. A. Dhasmana D. Jones B. Gabriel C. M. Sturman S. Davies N. W. S.. (2018). Human T-cell lymphotropic virus (HTLV)-associated encephalopathy: an under-recognised cause of acute encephalitis? Case series and literature review. J. Neurol. 265, 871–879. 10.1007/s00415-018-8777-z, PMID: 29423617 PMC5878187

[ref11] Cubie H. A. (2013). Diseases associated with human papillomavirus infection. Virology 445, 21–34. 10.1016/j.virol.2013.06.007, PMID: 23932731

[ref12] Doris K. Ashall J. Zuckerman M. Almond N. Anderson R. (2015). “Development of a lyophilized viral multiplex PCR run control reagent for clinical diagnoses” in *ECCMID* (Copenhagen), EV0904.

[ref13] Edridge A. W. D. Deijs M. van Zeggeren I. E. Kinsella C. M. Jebbink M. F. Bakker M.. (2019). Viral metagenomics on cerebrospinal fluid. Genes (Basel) 10:332. 10.3390/genes10050332, PMID: 31052348 PMC6562652

[ref14] Eibach D. Hogan B. Sarpong N. Winter D. Struck N. S. Adu-Sarkodie Y.. (2019). Viral metagenomics revealed novel betatorquevirus species in pediatric inpatients with encephalitis/meningoencephalitis from Ghana. Sci. Rep. 9:2360. 10.1038/s41598-019-38975-z, PMID: 30787417 PMC6382885

[ref15] Feehery G. R. Yigit E. Oyola S. O. Langhorst B. W. Schmidt V. T. Stewart F. J.. (2013). A method for selectively enriching microbial DNA from contaminating vertebrate host DNA. PLoS One 8:e76096. 10.1371/journal.pone.0076096, PMID: 24204593 PMC3810253

[ref16] Florance N. R. Davis R. L. Lam C. Szperka C. Zhou L. Ahmad S.. (2009). Anti-N-methyl-D-aspartate receptor (NMDAR) encephalitis in children and adolescents. Ann. Neurol. 66, 11–18. 10.1002/ana.21756, PMID: 19670433 PMC2826225

[ref17] Fok A. Mateevici C. Lin B. Chandra R. V. Chong V. H. T. (2015). Encephalitis-associated human metapneumovirus pneumonia in adult, Australia. Emerg. Infect. Dis. 21, 2074–2076. 10.3201/eid2111.150608, PMID: 26488420 PMC4622250

[ref18] Gable M. S. Gavali S. Radner A. Tilley D. H. Lee B. Dyner L.. (2009). Anti-NMDA receptor encephalitis: report of ten cases and comparison with viral encephalitis. Eur. J. Clin. Microbiol. Infect. Dis. 28, 1421–1429. 10.1007/s10096-009-0799-0, PMID: 19718525 PMC2773839

[ref19] Glaser C. A. Honarmand S. Anderson L. Schnurr D. P. Forghani B. Cossen C. K.. (2006). Beyond viruses: clinical profiles and etiologies associated with encephalitis. Clin. Infect. Dis. 43, 1565–1577. 10.1086/509330, PMID: 17109290

[ref20] Granerod J. Ambrose H. E. Davies N. W. Clewley J. P. Walsh A. L. Morgan D.. (2010a). Causes of encephalitis and differences in their clinical presentations in England: a multicentre, population-based prospective study. Lancet Infect. Dis. 10, 835–844. 10.1016/S1473-3099(10)70222-X, PMID: 20952256

[ref21] Granerod J. Cunningham R. Zuckerman M. Mutton K. J. Davies N. W. S. Walsh A. L.. (2010b). Causality in acute encephalitis: defining aetiologies. Epidemiol. Infect. 138:783. 10.1017/S0950268810000725, PMID: 20388231

[ref22] Granerod J. Tam C. C. Crowcroft N. S. Davies N. W. S. Borchert M. Thomas S. L. (2010c). Challenge of the unknown. A systematic review of acute encephalitis in non-outbreak situations. Neurology 75, 924–932. 10.1212/WNL.0b013e3181f11d65, PMID: 20820004

[ref23] Gurav Y. K. Tandale B. V. Jadi R. S. Gunjikar R. S. Tikute S. S. Jamgaonkar A. V.. (2010). Chandipura virus encephalitis outbreak among children in Nagpur division, Maharashtra, 2007. Indian J. Med. Res. 132, 395–399. PMID: 20966517

[ref24] Haley S. A. Atwood W. J. (2017). Progressive multifocal leukoencephalopathy: endemic viruses and lethal brain disease. Annu. Rev. Virol. 4, 349–367. 10.1146/annurev-virology-101416-041439, PMID: 28637388

[ref25] Hall R. J. Wang J. Todd A. K. Bissielo A. B. Yen S. Strydom H.. (2014). Evaluation of rapid and simple techniques for the enrichment of viruses prior to metagenomic virus discovery. J. Virol. Methods 195, 194–204. 10.1016/j.jviromet.2013.08.035, PMID: 24036074 PMC7113663

[ref26] Hannah M. J. Weiss U. Huttner W. B. (1998). Differential extraction of proteins from paraformaldehyde-fixed cells: lessons from synaptophysin and other membrane proteins. Methods 16, 170–181. 10.1006/meth.1998.0664, PMID: 9790863

[ref27] Hasan M. R. Rawat A. Tang P. Jithesh P. V. Thomas E. Tan R.. (2016). Depletion of human DNA in spiked clinical specimens for improvement of sensitivity of pathogen detection by Next-Generation Sequencing. J. Clin. Microbiol. 54, 919–927. 10.1128/JCM.03050-15, PMID: 26763966 PMC4809942

[ref28] Hoffmann B. Tappe D. Höper D. Herden C. Boldt A. Mawrin C.. (2015). A variegated squirrel bornavirus associated with fatal human encephalitis. N. Engl. J. Med. 373, 154–162. 10.1056/NEJMoa1415627, PMID: 26154788

[ref29] Jamur M. C. Oliver C. (2010). “Permeabilization of cell membranes” in Methods in molecular biology (Clifton, N.J), 63–66. 10.1007/978-1-59745-324-0_9 20012820

[ref30] Jmor F. Emsley H. C. Fischer M. Solomon T. Lewthwaite P. (2008). The incidence of acute encephalitis syndrome in Western industrialised and tropical countries. Virol. J. 5:134. 10.1186/1743-422X-5-134, PMID: 18973679 PMC2583971

[ref31] Kang Y. J. Zhou M. F. Huang W. Deng C. Yan G. Lu Z. H. (2017). Identification of a novel torque teno mini virus in cerebrospinal fluid from a child with encephalitis. Virol. Sin. 32, 541–544. 10.1007/s12250-017-4042-3, PMID: 29047018 PMC6598920

[ref32] Kawada J. -I. Okuno Y. Torii Y. Okada R. Hayano S. Ando S.. (2016). Identification of viruses in cases of pediatric acute encephalitis and encephalopathy using Next-Generation Sequencing. Sci. Rep. 6:33452. 10.1038/srep33452, PMID: 27625312 PMC5022051

[ref33] Kennedy P. G. E. Quan P. -L. Lipkin W. I. (2017). Viral encephalitis of unknown cause: current perspective and recent advances. Viruses 9:138. 10.3390/v9060138, PMID: 28587310 PMC5490815

[ref34] Kohl C. Brinkmann A. Dabrowski P. W. Radonić A. Nitsche A. Kurth A. (2015). Protocol for metagenomic virus detection in clinical specimens. Emerg. Infect. Dis. 21, 48–57. 10.3201/eid2101.140766, PMID: 25532973 PMC4285256

[ref35] Lewandowska D. W. Zagordi O. Zbinden A. Schuurmans M. M. Schreiber P. Geissberger F. -D.. (2015). Unbiased metagenomic sequencing complements specific routine diagnostic methods and increases chances to detect rare viral strains. Diagn. Microbiol. Infect. Dis. 83, 133–138. 10.1016/j.diagmicrobio.2015.06.017, PMID: 26231254 PMC7172999

[ref36] Li H. (2013). Aligning sequence reads, clone sequences and assembly contigs with BWA-MEM. *arXiv* [Preprint]. Available at: http://arxiv.org/abs/1303.3997 (Accessed April 12, 2019).

[ref37] Lin H. -H. Liao Y. -C. (2017). drVM: a new tool for efficient genome assembly of known eukaryotic viruses from metagenomes. GigaScience 6, 1–10. 10.1093/gigascience/gix003, PMID: 28369462 PMC5466706

[ref38] Lo Y. M. D. Tein M. S. C. Lau T. K. Haines C. J. Leung T. N. Poon P. M. K.. (1998). Quantitative analysis of fetal DNA in maternal plasma and serum: implications for noninvasive prenatal diagnosis. Am. J. Hum. Genet. 62, 768–775. 10.1086/301800, PMID: 9529358 PMC1377040

[ref39] Lyons J. L. Thakur K. T. Lee R. Watkins T. Pardo C. A. Carson K. A.. (2015). Utility of measuring (1,3)-β-d-glucan in cerebrospinal fluid for diagnosis of fungal central nervous system infection. J. Clin. Microbiol. 53, 319–322. 10.1128/JCM.02301-14, PMID: 25378578 PMC4290946

[ref40] Mailles A. Stahl J. (2009). Infectious encephalitis in France in 2007: a national prospective study. Clin. Infect. Dis. 49, 1838–1847. 10.1086/648419, PMID: 19929384

[ref41] Manso C. F. Bibby D. F. Mbisa J. L. (2017). Efficient and unbiased metagenomic recovery of RNA virus genomes from human plasma samples. Sci. Rep. 7:4173. 10.1038/s41598-017-02239-5, PMID: 28646219 PMC5482852

[ref42] McIntyre A. B. R. Ounit R. Afshinnekoo E. Prill R. J. Hénaff E. Alexander N.. (2017). Comprehensive benchmarking and ensemble approaches for metagenomic classifiers. Genome Biol. 18:182. 10.1186/s13059-017-1299-7, PMID: 28934964 PMC5609029

[ref43] Mehta R. Gerardin P. de Brito C. A. A. Soares C. N. Ferreira M. L. B. Solomon T. (2018). The neurological complications of chikungunya virus: a systematic review. Rev. Med. Virol. 28:e1978. 10.1002/rmv.1978, PMID: 29671914 PMC5969245

[ref44] Michael B. D. Sidhu M. Stoeter D. Roberts M. Beeching N. J. Bonington A.. (2010). Acute central nervous system infections in adults—a retrospective cohort study in the NHS North West region. QJM 103, 749–758. 10.1093/qjmed/hcq121, PMID: 20657024

[ref45] Miller S. Naccache S. N. Samayoa E. Messacar K. Arevalo S. Federman S.. (2019). Laboratory validation of a clinical metagenomic sequencing assay for pathogen detection in cerebrospinal fluid. Genome Res. 29, 831–842. 10.1101/gr.238170.118, PMID: 30992304 PMC6499319

[ref46] Morfopoulou S. Mee E. T. Connaughton S. M. Brown J. R. Gilmour K. Chong W. K.. (2017). Deep sequencing reveals persistence of cell-associated mumps vaccine virus in chronic encephalitis. Acta Neuropathol. 133, 139–147. 10.1007/s00401-016-1629-y, PMID: 27770235 PMC5209397

[ref47] Naccache S. N. Peggs K. S. Mattes F. M. Phadke R. Garson J. A. Grant P.. (2015). Diagnosis of neuroinvasive astrovirus infection in an immunocompromised adult with encephalitis by unbiased Next-Generation Sequencing. Clin. Infect. Dis. 60, 919–923. 10.1093/cid/ciu912, PMID: 25572898 PMC4345816

[ref48] Oechslin C. P. Lenz N. Liechti N. Ryter S. Agyeman P. Bruggmann R.. (2018). Limited correlation of shotgun metagenomics following host depletion and routine diagnostics for viruses and bacteria in low concentrated surrogate and clinical samples. Front. Cell. Infect. Microbiol. 8:375. 10.3389/fcimb.2018.00375, PMID: 30406048 PMC6206298

[ref49] Oyola S. O. Gu Y. Manske M. Otto T. D. O’Brien J. Alcock D.. (2013). Efficient depletion of host DNA contamination in malaria clinical sequencing. J. Clin. Microbiol. 51, 745–751. 10.1128/JCM.02507-12, PMID: 23224084 PMC3592063

[ref50] Palacios G. Druce J. Du L. Tran T. Birch C. Briese T.. (2008). A new arenavirus in a cluster of fatal transplant-associated diseases. N. Engl. J. Med. 358, 991–998. 10.1056/NEJMoa073785, PMID: 18256387

[ref51] Parras-Moltó M. Rodríguez-Galet A. Suárez-Rodríguez P. López-Bueno A. (2018). Evaluation of bias induced by viral enrichment and random amplification protocols in metagenomic surveys of saliva DNA viruses. Microbiome 6:119. 10.1186/s40168-018-0507-3, PMID: 29954453 PMC6022446

[ref52] Phan T. G. Messacar K. Dominguez S. R. da Costa A. C. Deng X. Delwart E. (2016). A new densovirus in cerebrospinal fluid from a case of anti-NMDA-receptor encephalitis. Arch. Virol. 161, 3231–3235. 10.1007/s00705-016-3002-9, PMID: 27522586 PMC6550996

[ref53] Phan T. G. Mori D. Deng X. Rajidrajith S. Ranawaka U. Fan Ng T. F.. (2015). Small circular single stranded DNA viral genomes in unexplained cases of human encephalitis, diarrhea, and in untreated sewage. Virology 482, 98–104. 10.1016/j.virol.2015.03.011, PMID: 25839169 PMC4461510

[ref54] Piantadosi A. Kanjilal S. Ganesh V. Khanna A. Hyle E. P. Rosand J.. (2018). Rapid detection of powassan virus in a patient with encephalitis by metagenomic sequencing. Clin. Infect. Dis. 66, 789–792. 10.1093/cid/cix792, PMID: 29020227 PMC5850433

[ref55] Quan P. L. Wagner T. A. Briese T. Torgerson T. R. Hornig M. Tashmukhamedova A.. (2010). Astrovirus encephalitis in boy with X-linked agammaglobulinemia. Emerg. Infect. Dis. 16, 918–925. 10.3201/eid1606.091536, PMID: 20507741 PMC4102142

[ref56] Scheer S. John R. M. (2016). Anti-N-methyl-D-aspartate receptor (NMDAR) encephalitis in children and adolescents. J. Pediatr. Health Care 30, 347–358. 10.1016/j.pedhc.2015.09.004, PMID: 26507948

[ref57] Schmieder R. Edwards R. (2011). Quality control and preprocessing of metagenomic datasets. Bioinformatics 27, 863–864. 10.1093/bioinformatics/btr026, PMID: 21278185 PMC3051327

[ref58] Schroeder K. Nitsche A. (2010). Multicolour, multiplex real-time PCR assay for the detection of human-pathogenic poxviruses. Mol. Cell. Probes 24, 110–113. 10.1016/j.mcp.2009.10.008, PMID: 19879351

[ref59] Schwartz U. Németh A. Diermeier S. Exler J. H. Hansch S. Maldonado R.. (2019). Characterizing the nuclease accessibility of DNA in human cells to map higher order structures of chromatin. Nucleic Acids Res. 47, 1239–1254. 10.1093/nar/gky1203, PMID: 30496478 PMC6379673

[ref60] Solomon T. Hart I. J. Beeching N. J. (2007). Viral encephalitis: a clinician’s guide. Pract. Neurol. 7, 288–305. 10.1136/jnnp.2007.129098, PMID: 17885268

[ref61] Solomon T. Michael B. D. Smith P. E. Sanderson F. Davies N. W. S. Hart I. J.. (2012). Management of suspected viral encephalitis in adults—association of British Neurologists and British Infection Association National Guidelines. J. Inf. Secur. 64, 347–373. 10.1016/j.jinf.2011.11.014, PMID: 22120595

[ref62] Tan L. V. van Doorn H. R. Nghia H. D. T. Chau T. T. H. Tu L. T. P. de Vries M.. (2013). Identification of a new cyclovirus in cerebrospinal fluid of patients with acute central nervous system infections. MBio 4, e00231–e00213. 10.1128/mBio.00231-13, PMID: 23781068 PMC3684831

[ref63] Thakur K. T. Motta M. Asemota A. O. Kirsch H. L. Benavides D. R. Schneider E. B.. (2013). Predictors of outcome in acute encephalitis. Neurology 81, 793–800. 10.1212/WNL.0b013e3182a2cc6d, PMID: 23892708 PMC3908458

[ref64] Thoendel M. Jeraldo P. R. Greenwood-Quaintance K. E. Yao J. Z. Chia N. Hanssen A. D.. (2016). Comparison of microbial DNA enrichment tools for metagenomic whole genome sequencing. J. Microbiol. Methods 127, 141–145. 10.1016/j.mimet.2016.05.022, PMID: 27237775 PMC5752108

[ref65] Tunkel A. R. Glaser C. A. Bloch K. C. Sejvar J. J. Marra C. M. Roos K. L.. (2008). The management of encephalitis: clinical practice guidelines by the Infectious Diseases Society of America. Clin. Infect. Dis. 47, 303–327. 10.1086/589747, PMID: 18582201

[ref66] Venkatesan A. Tunkel A. R. Bloch K. C. Lauring A. S. Sejvar J. Bitnun A.. (2013). Case definitions, diagnostic algorithms, and priorities in encephalitis: consensus statement of the international encephalitis consortium. Clin. Infect. Dis. 57, 1114–1128. 10.1093/cid/cit458, PMID: 23861361 PMC3783060

[ref67] Vidal L. R. de Almeida S. M. Cavalli B. M. Dieckmann T. G. Raboni S. M. Salvador G. L. O.. (2019). Human adenovirus meningoencephalitis: a 3-years’ overview. J. Neuro-Oncol. 25, 589–596. 10.1007/s13365-019-00758-7, PMID: 31102186

[ref68] Westbrook A. Ramsdell J. Schuelke T. Normington L. Bergeron R. D. Thomas W. K.. (2017). PALADIN: protein alignment for functional profiling whole metagenome shotgun data. Bioinformatics 33, 1473–1478. 10.1093/bioinformatics/btx021, PMID: 28158639 PMC5423455

[ref69] Wilson M. R. Sample H. A. Zorn K. C. Arevalo S. Yu G. Neuhaus J.. (2019). Clinical metagenomic sequencing for diagnosis of meningitis and encephalitis. N. Engl. J. Med. 380, 2327–2340. 10.1056/NEJMoa1803396, PMID: 31189036 PMC6764751

[ref70] Woolhouse M. E. J. Adair K. (2013). The diversity of human RNA viruses. Futur. Virol. 8, 159–171. 10.2217/fvl.12.129, PMID: 29503665 PMC5831953

[ref71] Woolhouse M. Scott F. Hudson Z. Howey R. Chase-Topping M. (2012). Human viruses: discovery and emergence. Philos. Trans. R. Soc. Lond. Ser. B Biol. Sci. 367, 2864–2871. 10.1098/rstb.2011.0354, PMID: 22966141 PMC3427559

